# Hepatic Lesions Detected after Mastectomy, in Breast Cancer Patients with Hepatitis Background May Need to Undergo Liver Biopsy to Rule Out Second Primary Hepatocellular Carcinoma

**DOI:** 10.1371/journal.pone.0139782

**Published:** 2016-01-14

**Authors:** Qi-wen Chen, Hai-jin Li, Ya-nan Chen, Zhou-yu Ning, Song Gao, Ye-hua Shen, Zhi-qiang Meng, Sonya Vargulick, Bi-yun Wang, Hao Chen

**Affiliations:** 1 Department of Integrative Oncology, Fudan University Shanghai Cancer Center, Shanghai, China; 2 Department of Oncology, Shanghai Medical College, Fudan University, Shanghai, China; 3 Department of Medical Oncology, Shaoxing Hospital, China Medical University, Shaoxing, China; 4 Department of Medical Oncology, Fudan University Shanghai Cancer Center, Shanghai, China; 5 Department of Pharmacy, Albany College of Pharmacy and Health Sciences, Albany, New York, United States of America; Yonsei University College of Medicine, REPUBLIC OF KOREA

## Abstract

**Purpose:**

Liver metastasis is a common phenomenon in breast cancer patients. Hepatic lesions detected in breast cancer patients may be easily misdiagnosed as metastatic sites, rather than being treated as primary foci. This descriptive study aims to investigate the clinicopathological characteristics of second primary hepatocellular carcinoma in breast cancer patients and to infer in which circumstances liver biopsy is needed.

**Methods:**

Eighty-one consecutive breast cancer patients with hepatic lesions admitted to our department were retrospectively studied and analyzed from January 2009 to March 2014 according to Warren and Gates’ criteria for second primary cancers.

**Results:**

Second primary hepatocellular carcinoma was observed in sixteen of seventy eight patients with breast cancer. There was a significant difference in HBV status between the second HCC group and liver metastases group (*P<0*.*0001*). There was no significant difference in age (*P = 0*.*2254*) and family history (*P = 0*.*1160*) between second primary HCC and metastases group. Two of these patients had synchronous second primary hepatocellular carcinoma and the remaining fourteen patients had metachronous second primary HCC. All sixteen patients were infected with hepatitis, including hepatitis virus B and C, or resolved HBV infection.

**Conclusions:**

Breast cancer patients with either HBV infection or resolved HBV infection, regardless of an elevated AFP level, may receive liver biopsy to avoid unnecessary and inappropriate treatments for metastasis. Awareness of second primary HCC in breast cancer patients needs to be emphasized.

## Introduction

Breast cancer is the most common malignancy among women worldwide [1]. During the last twenty years, the advancement in surgery, chemotherapy, radiology, and endocrine therapy has greatly improved the prognosis and life expectancy of breast cancer patients, exempting it from the leading cancer-related causes among women. [2]. Accordingly, this tendency has led to an obvious increase in the number of breast cancer patients who are at risk of developing second primary cancers[3–5]. As reported in 2002, women with breast cancer consisted of approximately 25% of the total multiple cancer patients in the United States [6]. Meanwhile, several studies were conducted aiming to ascertain those risks [7–9]. Among these studies, some malignant tumors, including lung cancer, esophageal cancer, leukemia and soft tissue tumor were identified [7–9]. However, for several other malignant tumors, the results are inconclusive and second primary HCCs have not been well revealed or put due emphasis on in the treatment for breast cancers.

If a lesion on the stomach, intestine, or other uncommon metastatic organ was detected during the follow-up in breast cancer patients, it would easily be recognized as a second primary cancer. Tumor cells often disseminate from breast to liver, one of the most metastatic site [1]. Therefore, when a hepatic lesion is detected in patients with a medical history of breast cancer, it may be considered as a metastatic site, not second primary liver cancer. As a consequence, these patients would receive inappropriate treatments such as intravenous chemotherapy and endocrine therapy, which was mainly targeted at tumors in the breast, thus missing the appropriate time to treat primary liver cancer. Thus, definitive diagnosis is the first step toward a new treatment regimen.

Distinguishing a second primary cancer from recurrent cancer or metastatic lesions can sometimes be problematic. The 2013 NCCN guidelines for breast cancer doesn’t clearly state whether hepatic lesions detected in breast cancer patients should undergo liver biopsy to rule out second primary liver cancer. However, not all hepatic lesions in breast cancer patients are metastatic sites [10]. Thus, reckless judgment should be avoided and reevaluation of the hepatic lesion is necessary. This descriptive study aims to investigate the clinicopathological characteristics of the second primary hepatocellular carcinoma in breast cancer patients and to infer in which circumstances liver biopsy is needed.

## Patients and Methods

This retrospective study was approved by the ethics committee of Shanghai Cancer Center, Fudan University. Upon admission, written informed consent was obtained from all the patients. Their clinical data were collected for the future study. The retrospective study covered a time span of more than five years, from January 2009 to March 2014. Eighty-one patients consecutive breast cancer patients with hepatic lesions admitted to our department were studied and analyzed. Breast cancer was diagnosed and confirmed by postoperative pathology. Three patients were excluded from this study due to the absence of cytomorphological or histological findings of the hepatic lesion. Therefore, the remaining seventy-eight patients who had undergone ultrasound-guided fine needle biopsy and had pathological diagnosis were included in this study. All seventy-eight patients had pathological diagnosis on their hepatic lesions. The patients were all investigated through abdominal imaging (ultrasonography, CT and MRI, or PET-CT when needed) and tumor markers was detected every three months. Among these seventy-eight patients, sixteen were finally diagnosed with second primary HCC and sixty-two liver metastases pathologically. Clinical properties, operative findings and pathological results were extracted from medical records from the second primary HCC patients. All imaging reports were administered and reviewed by two diagnostic readiologists.

### Criteria for diagnosis

In this study, the ultimate diagnosis of second HCC and liver metastases were determined according to pathological criteria of malignancy. Second primary cancer was determined according to Warren and Gates criteria[9]. It is required that the liver tissues should be histologically malignant in accordance with both criteria. Additionally, a minimum of 2cm-sized tumor-free tissue should be included in the lesions, and it should also be confirmed that the second cancer is not a metastatic or invasive site from the primary cancer. It is defined as a synchronous cancer if diagnosed simultaneously with the primary cancer or within the six months, while it is defined as a metachronous cancer if diagnosed six months after the primary cancer.

Statitical methods: T-test was used to analyze the age between the second HCC and the metastasis group. Chi-square test was used to analyze the HBV status, family history, cirrhosis, Ca153, radiotherapy, chemotherapy and endocrine therapy between the two goups. P<0.05 was considered statistically significant.

## Results

### General condition

Second primary hepatocellular carcinoma was observed in sixteen of seventy eight patients with breast cancer (20.5%). All the sixteen patients were female. The characteristics of the first primary breast cancer and postoperative treatments are shown in Table 1. The mean age at the detection of the first primary breast cancer was 52.4±1.9 years and the mean age at the detection of the second primary HCC was 55.4 ± 2.2 years. Two of these patients developed synchronous cancer, both of who had the second primary HCCs detected five months after the detection of breast cancer. The remaining fourteen patients had metachronous second primary HCC, with a mean time interval between the detections of the two primary cancers of 42.5±8.1 months, as shown in Table 2. Among the sixteen patients, twelve had family history of malignant tumors, while the other four patients denied family history of malignancy, as shown in Table 1. None of these patients have alcohol or smoking abuse. A statistically significant difference in HBV status was found between the second HCC and liver metastasis group(*P<0*.*0001*). There was no statistically significant difference detected in the age (*P = 0*.*2254*) and family history (*P = 0*.*1160*) between the two groups, which was shown in Table 3. Two of the patients who were ultimately diagnosed as HCC had typical imaging features of “fast in and fast out”, while the other fourteen second HCC patients had no such typical imaging feature. None of the patients in the metastasis group had typical HCC imaging features.

**Table 1:** The characteristics of the first primary breast cancer and postoperative treatments.

Case No.	Age*	Pathology	TNM	Postoperative treatments	Family history of malignancy
1	54	IDC, Lumina	T1N1M0	FEC, Docetaxel, Endocrine therapy	None
2	51	IDC, Lumina	T2N0M0	EC	Esophageal
3	51	IDC, TNBC	T2N0M0	PC, radiotherapy	None
4	53	IDC, Lumina	T1N0M0	Endocrine therapy	HCC
5	52	IDC, Lumina	T2N0M0	EC, endocrine therapy	HCC
6	55	IDC, TNBC	T2N1M0	TEC, radiotherapy	None
7	56	IDC, Lumina	T1N1M0	FEC, Docetaxel, Endocrine therapy	Esophageal
8	52	IDC, Her-2+	T2N1M0	FEC, paclitaxel, Herceptin, radiotherapy	HCC
9	50	IDC, Lumina	T2N1M0	FEC, radiotherapy, endocrine therapy	Ovarian
10	50	IDC, Her-2+	T2N1M0	EC, paclitaxel, radiotherapy, endocrine therapy	Gastric
11	54	IDC, Her-2+	T2N0M0	Herceptin	HCC
12	53	IDC, TNBC	T1N1M0	TEC	Lung
13	50	IDC, Lumina	T2N1M0	FEC, paclitaxel	HCC
14	51	IDC, Her-2+	T1N0M0	TEC	Lymphoma
15	52	IDC, Lumina	T1N0M0	Endocrine therapy	None
16	55	IDC, TNBC	T2N0M0	FEC, radiotherapy	Cervical

Abbreviations: IDC, invasive ductal carcinoma; TNBC, triple negative breast cancer; FEC, 5-Fu, epirubicin, cyclophosphamide; EC, epirubicin, cyclophosphamide; PC, paclitaxel, carboplatin; TEC, paclitaxel, epirubicin, cyclophosphamide

*Age at the detection of the first primary breast cancer.

**Table 2:** Clinicopathological characteristics of the second primary HCC and applied treatment methods.

Case No.	Age*	Time interval# (months)	Differentiated degree	Cirrhosis	BCLC stage
1	57	38	Well	Yes	A
2	55	47	Poor	No	A
3	51	5	Moderate	Yes	A
4	58	55	Poor	Yes	C
5	56	53	Well	No	A
6	58	43	Well	No	A
7	56	5	Moderate	No	A
8	56	51	Well	No	A
9	53	32	Well	No	A
10	53	40	Well	Yes	A
11	57	42	Poor	Yes	A
12	56	38	Moderate	No	A
13	53	37	Well	Yes	A
14	54	35	Well	No	A
15	55	32	Well	No	A
16	59	54	Poor	No	A

Abbreviations: BCLC, Barcelona Clinic Liver Cancer

*Age at the detection of the second primary HCC

#time interval between the first primary breast cancer and second primary HCC.

**Table 3:** Comparison of HBV status, cirrhosis, family history and age between the two groups.

	Second HCC	Metastasis	ᵡ^2^	*P*
Hepatitis B				
No	0	56	N/A	<0.0001*
Yes	16	6		
Family history				
No	4	29	2.47	0.1160
Yes	12	33		
Cirrhosis				
No	10	61	20.05	<0.0001
Yes	6	1		
Age (mean,SD)	55.4(2.2)	52.2(10.4)	1.22**	0.2254
Ca153				
Elevated	2	5	N/A	0.6277*
Normal	14	57		
Radiotherapy				
Yes	6	27	0.02	0.8785
No	10	35		
Chemotherapy				
Yes	13	54	N/A	0.6870*
No	3	8		
Endocrine- therapy				
Yes	7	30	0.0025	0.9589
No	9	32		

* fisher’s exact test

** t value

### Clinicopathological characteristics of the second primary HCC and applied treatment approaches

**Fig 1:**
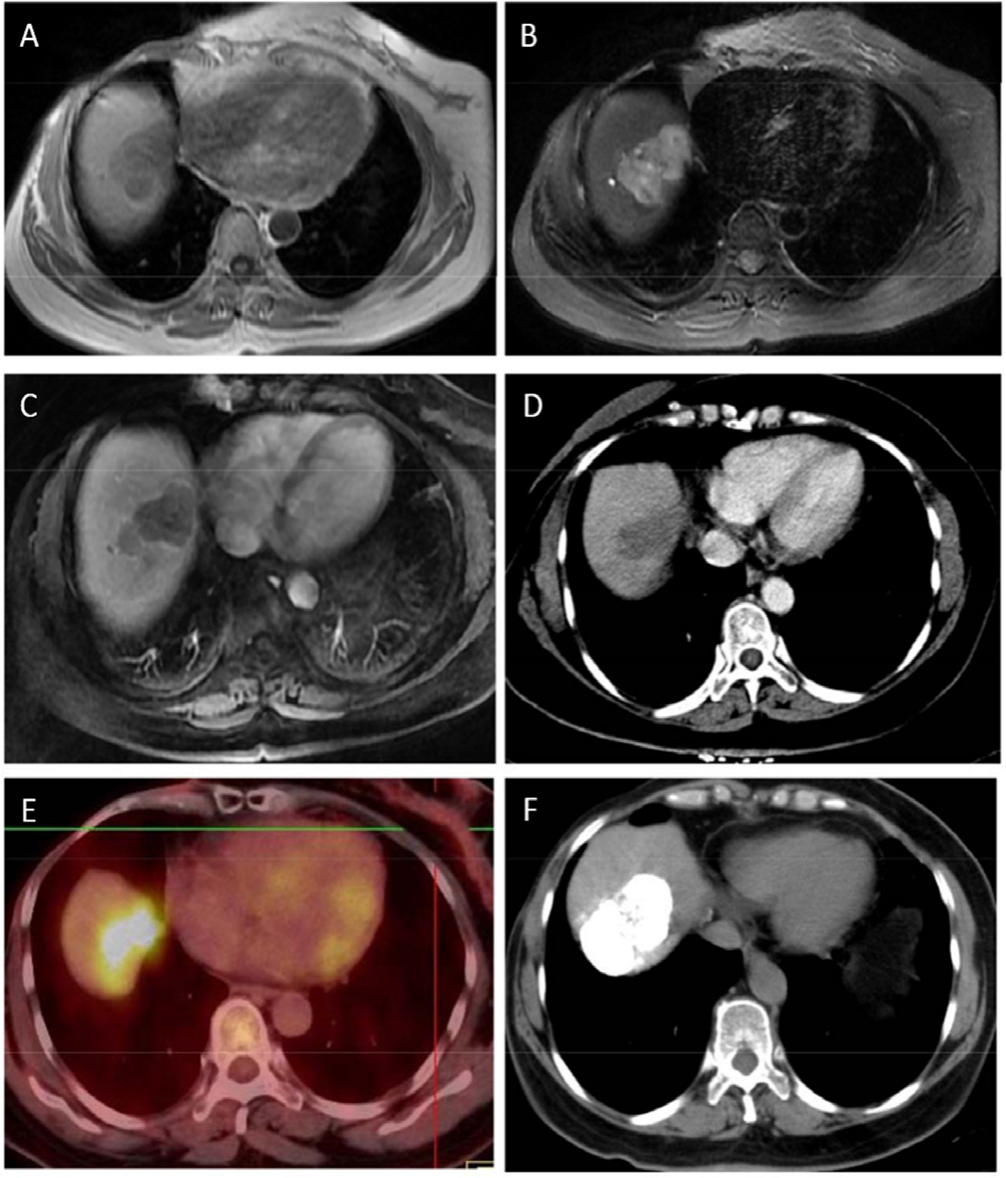
A: T1 weighted MR image shows irregular and clear border of the lesion with low signal. B: T2 weighted MR image shows high signal of the lesion with inhomogeneous signal inside. C: Late arterial phase of dynamic contrast enhanced scan shows central lesion without obvious enhancement, but part of the border has ribbon-like enhancement. D: Portal venous phase of dynamic contrast enhanced scan shows central lesion still has no enhancement and the previous enhancement part hasn’t washed out. E: PET-CT shows abnormal increase of radioactivity uptake with SUV max 6.9. F:CT scan shows good lipiodol deposition after transarterial chemoembolization.

**Fig 2:**
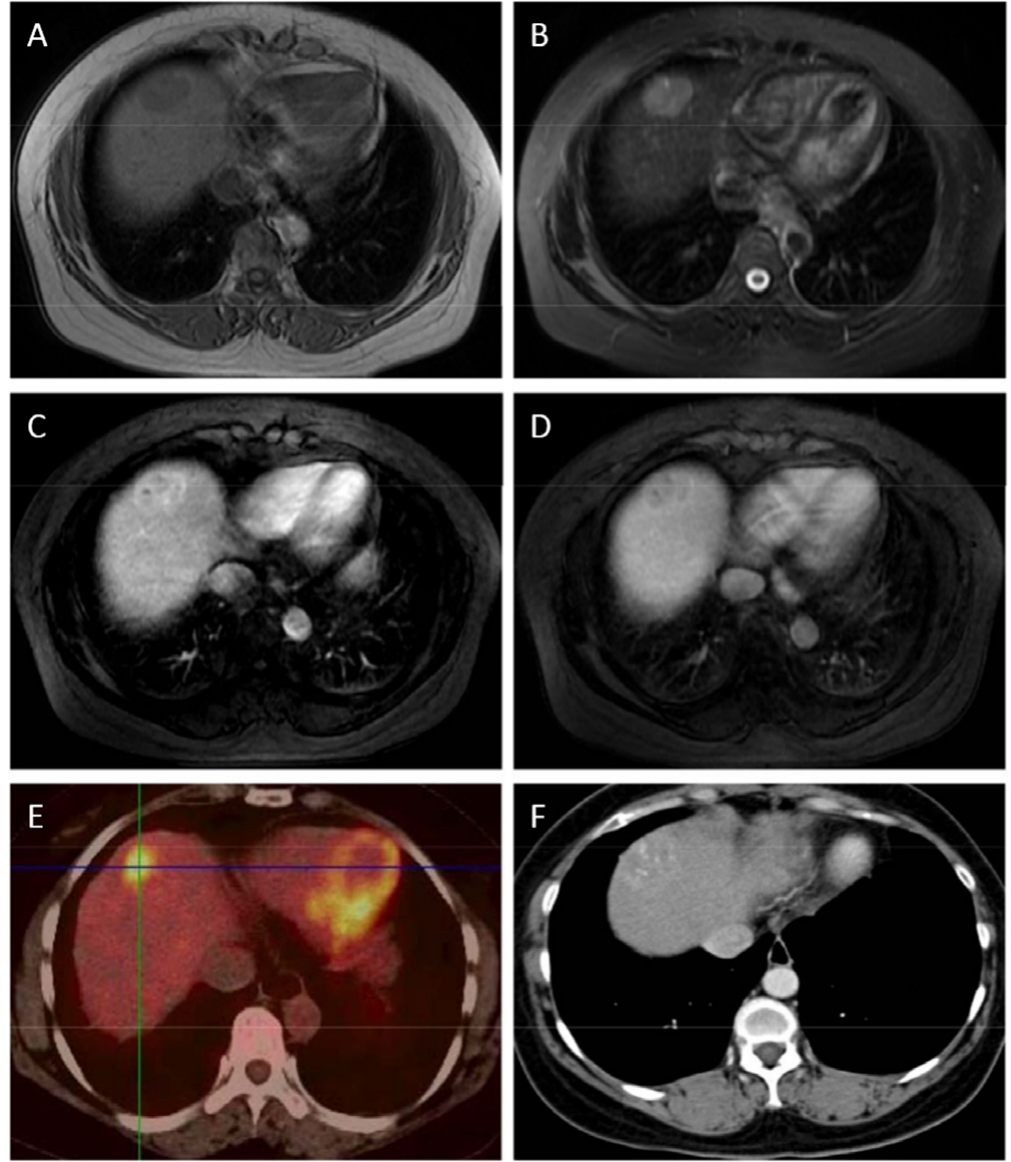
A: A 2.9*2.2cm sized round lesion is seen in the segment 8 of the liver which shows low signal intensity on T1-weighted MR image. B: A 2.9*2.2cm sized round lesion is seen in the segment 8 of the liver which shows slightly high signal intensity on T2-weighted MR image. C: Late arterial phase of dynamic contrast enhanced scan shows inhomogeneous enhancement in the lesion with obvious enhancement on the border. D: Portal venous phase of dynamic contrast enhanced scan shows the overall enhancement has not washed out. E: PET-CT reviews: increasing abnormal uptake with SUV max 6.8. F: CT scan shows punctate distribution lipiodol deposition inside the lesion after TACE.

Fourteen patients had only one hepatic lesion, with eleven on the right lobe and three on the left lobe of the liver. One patient had three lesions on both lobes, who was the only one detected with portal vein tumor thrombus and in BCLC stage C. The remaining patients had two lesions on the right lobe. The characteristics of the hepatic lesions were shown in Table 4. Degrees of differentiation vary among them and were shown in Table 2, with nine well differentiated, three moderately differentiated and four poorly differentiated. The imaging features in this study were as follows: single lesion without cirrhosis background, necrotic or cystic lesions, and mild edge enhancement in artery phase, which were difficult to distinguish from metastasis. The MRI, CT, and PET-CT of the lesions in two of the patients were shown in Fig 1 and Fig 2. No significant abnormalities were detected in the liver function of these patients. Surgery, radio frequency ablation, and transarterial chemoembolization were the main therapies for these patients according to their BCLC stages and patients’ self will.

**Table 4:** Characteristics of the hepatic lesions.

Case No.	Location	Single/Multiple	Size (mm*mm)	PVTT	Treatments
1	Right lobe	Single	32*42	No	Surgery
2	Right lobe	Single	31*51	No	RFA
3	Right lobe	Single	32*22	No	RFA
4	Both lobes	Three	54*40,36*33,33*30	Yes	TACE+RFA+Sorafinib
5	Left lobe	Single	52*40	No	TACE+RFA
6	Right lobe	Single	47*44	No	TACE+RFA
7	Right lobe	Single	24*18	No	Surgery
8	Right lobe	Two	29*22,36*32	No	RFA
9	Right lobe	Single	56*47	No	TACE+RFA
10	Left lobe	Single	48*39	No	TACE+RFA
11	Right lobe	Single	47*38	No	TACE+RFA
12	Right lobe	Single	43*38	No	Surgery
13	Right lobe	Single	35*34	No	Surgery
14	Left lobe	Single	27*24	No	RFA
15	Right lobe	Single	41*39	No	TACE+RFA
16	Right lobe	Single	59*55	No	TACE+RFA

Abbreviation: PVTT, portal vein tumor thrombus; TACE, transcatheter arterial chemoembolization; RFA, radiofrequency ablation

### HBV status

There was significant statistical difference in HBV status between the second HCC group and metastasis group. In the second primary HCC group, a total of seven patients had chronic hepatitis B infection, while seven patients had resolved hepatitis B infection. And one patient was an inactive HBsAg carrier while one patient suffered from hepatitis C infection. However, only three patients received anti-viral medication before the diagnosis of HCC. Among the three patients, two started lamivudine before the confirmation of breast cancer and one received entecavir during the postoperative chemotherapy without test for HBV profile regularly. The remaining thirteen patients received no medication for their hepatitis. The HBV status of these sixteen patients was shown in Table 5. Meanwhile, elevated AFP levels were observed in only four patients(25%), while the other twelve patients’ AFP levels were within the normal range. Regretfully, due to the lack of prior data on HBV status and HBV DNA copies, changes in HBsAg and HBV copies cannot be extracted and compared in this study. But elevated HBV DNA copies were detected in seven patients at the diagnosis of second primary HCC. Additionally, liver functions were monitored and compared before, during and after the treatment of breast cancer, without apparent abnormality detected. Thus, there is no sufficient evidence to prove HBV reactivations occurred in these patients.

**Table 5:** HBV status at the diagnosis of second primary HCC and prior anti-viral treatment.

Case No.	HBsAg	HBsAb	HBeAg	HBeAb	HBcAb	HBV-DNA	AFP(ng/ml)	Prior ant-viral treatment
1	+	-	+	-	+	5.76E5	957.10	Lamivudine
2	+	-	-	+	+	4.54E4	3.31	None
3	+	-	-	+	+	5.43E5	9.07	Lamivudine
4	+	-	-	+	+	2.45E4	3.20	Entecavir
5	-	+	-	+	+	_	1.24	None
6	-	+	-	+	+	_	4.18	None
7	-	-	-	-	+	_	8.25	None
8	-	-	-	-	+	_	4.49	None
9	-	+	-	+	+	_	46.28	None
10	+	-	-	+	+	3.18E4	5.35	None
11	+	-	+	-	+	5.29E5	15.89	None
12	-	-	-	-	+	_	7.93	None
13	+	-	-	+	+	3.47E6	80.83	None
14	-	-	-	-	+	_	1.99	None
15	-	-	-	-	+	_	5.87	None
16	+	-	-	+	+	_	6.14	None

Abbreviation: HBV, hepatitis B virus; AFP, alpha fetoprotein

## Discussion

### Why did these patients suffer from second primary HCC?

It is known that patients who suffer from cancers may have a 20% higher risk of subsequent primary malignant tumors[11–12]. Breast cancer is both prevalent in women, and the most commonly occurred multiple malignancy [6]. In recent years, advances in the treatment for breast cancer have resulted in better outcomes and prolonged life expectancy among these women, leading to a marked increase in the number of survivors who are more susceptible to developing second primary cancers, including genital, esophageal, salivary gland cancers and sarcoma, either due to treatment-related or other factors[7–9]. Until now, there have been no studies explicitly demonstrating any treatment-related correlation between breast cancer and second primary HCC, neither experimentally nor clinically. Similarly, in our study, the patients received a variety of treatment, which made it difficult to distinguish if a specific treatment modality caused the second primary HCC.

However, we do not regard the occurrence of second primary HCC as a coincidence. Hepatitis B is endemic and the predominant cause of HCC in China [13]. Many Chinese patients who suffered from a first primary cancer are either HBV carriers or infected with HBV, which may result in cirrhosis after some years. This is also no exception to breast cancer survivors. It is known that most of the HCC patients in China have underlying cirrhosis[14]. However, it was unexpected to find that cirrhosis was detected in only six patients (37.5%) in the sixteen cases, suggesting cirrhosis was not a major cause for those patients to develop second primary HCC.

### HBV status: not only chronic HBV infection but also resolved HBV infection may play a role in second primary HCC in breast cancer survivors

In this study, it can be easily noticed that all the patients suffering from second primary HCC had abnormalities in their hepatitis profile, with seven patients suffering from chronic hepatitis B infection, one patient suffering from hepatitis C infection, and eight patients having resolved HBV infection as shown in Table 4. In the treatment for breast cancer, chemotherapy may aggravate liver damage, enhance the replication of hepatitis virus and even possibly reactivate the hepatitis B virus, thus accelerating the development of the status of the disease from hepatitis B or cirrhosis to HCC. Therefore, anti-viral therapy is essential as a preemptive treatment for some breast cancer patients with HBV infection while receiving chemotherapy.

Recently, there have been a wide variety of reports on hepatitis B virus reactivation in non-Hodgkin’s lymphoma patients treated with Rituximab[15–17]. Other than lymphoma, there are also hepatitis B reactivations in solid tumors which can be found in breast cancer patients undergoing chemotherapy containing epirubicin[18]and HCC patients undergoing TACE[19], etc.

Unfortunately, due to the lack of prior data on HBV status and HBV DNA copies during the treatment for breast cancer, the changes of HBsAg and HBV copies could not be extracted and compared in this study. Thus, it could not be determined whether HBV was reactivated in these patients. However, liver functions were monitored and compared before, during and after the treatment for breast cancer, without evidence of apparent abnormality detected. This may suggest there was no HBV reactivation in these sixteen patients, because most HBV reactivated patients undergo an obvious elevation in alanine transaminase.

In this study, three of the sixteen patients received anti-viral medication before the treatments for breast cancer, but unfortunately still developed second HCC. It has been well established that anti-viral medication can decrease the incidence of HCC in HBV infected patients[20] However, whether it can exert its effect in preventing second primary HCC in breast cancer patients is yet unknown. Therefore, a prospective study with a larger sample is needed to further verify this assumption. But we do suggest that HBV DNA level and HBV profile should be put more emphasis on during the chemotherapy, and anti-viral medication should be provided to the patients when necessary, in order to prevent HBV reactivation and potential second primary HCC.

### Age and family history of malignancy

One study [21] has reported that patients diagnosed with breast cancer at an earlier age (<50) are more susceptible to developing second cancers later in life. But the same study contradictorily showed all sixteen patients detected with first primary breast cancer and second primary HCC at more than fifty years of age. Meanwhile, among the sixteen patients who developed second primary HCC in our study, twelve patients (75%) had family history of cancers, with five HCC, two esophageal, and one gastric, ovarian, cervical, lung and lymphoma. Some occurrences of the second primary cancers may be due to the genetic mutations which are jointly shared by the primary and the second one [22]. It has been known that patients of hereditary breast and ovarian cancer syndrome (HBOC), caused by a germline mutation in BRCA1 or BRCA2, have an increased risk for breast, ovarian, prostate and pancreatic cancers [23]. However, there are no persuasive laboratory evidences demonstrating joint genetic mutations or genetic susceptibility in the occurrence of breast cancer and HCC currently.

However, there was no significant difference detected in the age and family history between the second HCC group and liver metastasis group in this study. The results regarding age and family history of patients may be specifically relevant to HBV-related HCC, not the age or family history themselves. Meanwhile, according to current epidemiology data [24], the patients elder than fifty years of age and with family history seem to be at a greater risk of developing HCC. Therefore, it is suggested that age and family history not be ignored as risk factors in developing second HCC deapite no significant difference detected in age and family history between the two groups.

### Is a hepatic lesion detected in breast cancer patients a metastasis or second primary HCC?

Liver is a common site for metastasis. The incidence of liver metastasis in breast cancer patients is much higher than that in second primary liver cancer [9]. Thus, a hepatic lesion detected in breast cancer is more likely to be regarded as metastasis rather than second primary liver cancer. Based on our small yet representative samples, it may suggest that breast cancer patients with a history of hepatitis virus infection would be at a greater risk of developing second primary HCC when solitary hepatic lesions are detected during the follow-up. Therefore, metastasis should not be merely taken into account under such circumstances.

If a newly emerged lesion on the stomach, intestine, pancreas or other uncommon metastatic organ was detected during the follow-up in breast cancer patients, it would be easily determined as a second primary cancer. However, the liver is one of the most commonly known metastatic organs from breast cancer cells [1]. When a hepatic lesion is detected in patients with a medical history of breast cancer, it may be firstly considered as a metastatic site, not a second primary liver cancer. This may further leads to inappropriate treatments such as intravenous chemotherapy and endocrine therapy targeted at advanced breast cancer, thus missing the appropriate time to treat second primary HCC. Therefore, a definitive diagnosis is the first step toward a new treatment regimen.

The 2013 NCCN guidelines for breast cancer does not clearly state whether hepatic lesions detected in breast cancer patients should undergo liver biopsy to rule out second primary liver cancer. Besides, fearful of the complications resulting from liver biopsy, many doctors may be unwilling to perform routine liver biopsy for every patient. According to AASLD guidelines 2011[25], HCC can be clinically diagnosed based on hepatitis infection and characteristic imaging findings. This noninvasive method avoids unnecessary liver biopsy in some cases, however, it would easily cause predicament in the diagnosis for those patients without elevated AFP levels or typical imaging manifestations. In this study, most of the patients had no elevated AFP levels or typical imaging manifestations, which could be misdiagnosed as metastasis without liver biopsy.

It is known that the typical imaging features for HCC includes: pseudocapsule, arterial enhancement with delayed washout, portal vein invasion and tumor thrombi formation [26]. However, these characteristics were not typically manifested in this group of patients. The imaging features in our study are mainly as follows: single lesion without cirrhosis background, necrotic or cystic lesions, and mild edge enhancement in artery phase. In the cases with single hepatic lesion and without baseline cirrhosis, the presentation of cystic or necrotic lesion with inapparent enhancement in arterial phase may be difficult to distinguish from bull’s eye sign and can be easily mistaken as liver metastasis. Currently, metastatic liver cancer may be the primary consideration for those patients with a previous history of breast cancer by many clinicians. Furthermore, metastatic liver cancer may present with various morphological features as well as blood perfusion and enhancement pattern, which can further increase the challenge for the differential diagnosis from nodular liver cancer, mixed liver cancer, highly differentiated HCC and HCC with cystic degeneration or halo sign. Therefore, it is necessary for these patients to undergo liver biopsy under such conditions.

### Why we advocate performance of fine needle biopsy on these patients?

Although HCC could be diagnosed via non-invasive approach in some cases, imaging finding is not completely precise and reliable. Undoubtedly, pathological diagnosis is the golden standard which can achieve nearly 100% accuracy while providing detailed information on hormone receptors of breast cancer, which may be helpful for the subsequent treatment for breast cancer patients with liver metastasis. Meanwhile, according to our formerly published work [27], fine needle biopsy is a both safe and low cost minimally invasive approach. Therefore, patients should receive fine needle biopsy if they have no apparent contraindications. However, we assume that some HCC patients with typical imaging features could be diagnosed via non-invasive approaches. Liver biopsy could be avoided if a nodule shows the typical enhancement pattern for HCC in patients with both chronic HBV infection or cirrhosis, and prior breast cancer history.

### Limitation of this study

The incidence of second HCC in this study was 20.5%(16/78), which is much higher than that reported in epidemiological study. This was mainly due to the selection bias in this study for the reason that our department mainly focuses on the hepato-biliary cancers. Some of the included patients in this study were recommended by the oncologists from other departments. So these samples had been pre-selected, which may cause the selection bias. However, it should be noted that it was not designed purposely. Meanwhile, due to the low incidence, it is uncommon that a breast cancer patient develops second HCC. Besides, the small sample may render the statistical analysis not as convincing as the result form a larger one. Unlike most of the epidemiologic studies previously published on multiple primary cancers, this study is more inclined to be a descriptive one and delineates a phenomenon to give some suggestions to the doctors when handling such cases, rather than merely offering precise statistical results. In general, the selection bias and small sample would not affect the result and conclusion of this descriptive study.

In this study, there were no patients who had second primary HCC without hepatitis infection. In the study, HCCs patients seemed to be mainly associated with HBV infection, whether a patient had cirrhosis or not. Therefore, the conclusions from the study are specifically targeted at breast cancer with HBV carriers, and cannot be generalized in all spectrums of breast cancer patients.

## Conclusion

Hepatic lesions detected in patients who previously suffered from breast cancer with either HBV infection or a resolved HBV infection, regardless of AFP levels, may receive liver biopsy to avoid unnecessary and inappropriate treatments for metastasis. Awareness of second primary HCC in breast cancer patients needs to be made by clinicians.
